# Automatic Segmentation of Lung Carcinoma Using 3D Texture Features in 18-FDG PET/CT

**DOI:** 10.1155/2013/980769

**Published:** 2013-02-26

**Authors:** Daniel Markel, Curtis Caldwell, Hamideh Alasti, Hany Soliman, Yee Ung, Justin Lee, Alexander Sun

**Affiliations:** ^1^Medical Physics Unit, University of McGill, Montreal, QC, Canada H3A 0G4; ^2^Department of Radiation Oncology, Odette Cancer Centre, the Sunnybrook Health Sciences Centre, Toronto, ON, Canada M4N 3M5; ^3^Department of Radiation Oncology, Princess Margaret Hospital, Toronto, ON, Canada M5G 2M9

## Abstract

Target definition is the largest source of geometric uncertainty in radiation therapy. This is partly due to a lack of contrast between tumor and healthy soft tissue for computed tomography (CT) and due to blurriness, lower spatial resolution, and lack of a truly quantitative unit for positron emission tomography (PET). First-, second-, and higher-order statistics, Tamura, and structural features were characterized for PET and CT images of lung carcinoma and organs of the thorax. A combined decision tree (DT) with K-nearest neighbours (KNN) classifiers as nodes containing combinations of 3 features were trained and used for segmentation of the gross tumor volume. This approach was validated for 31 patients from two separate institutions and scanners. The results were compared with thresholding approaches, the fuzzy clustering method, the 3-level fuzzy locally adaptive Bayesian algorithm, the multivalued level set algorithm, and a single KNN using Hounsfield units and standard uptake value. The results showed the DTKNN classifier had the highest sensitivity of 73.9%, second highest average Dice coefficient of 0.607, and a specificity of 99.2% for classifying voxels when using a probabilistic ground truth provided by simultaneous truth and performance level estimation using contours drawn by 3 trained physicians.

## 1. Introduction

 The clinical outcome of radiation therapy is closely linked to the ability to deliver dose within tightly confined boundaries to maximize target control while minimizing dose to surrounding tissue to reduce the probability of complications. This is the basis for treatment planning in radiotherapy, the success of which relies on the minimization of geometric and dosimetric uncertainties. Target uncertainty is compounded by movement when disease is present in the thorax which can be minimized with the aid of breath hold techniques such as audio coaching or active breathing control using a frame. However, for many patients who suffer from breathing difficulties, these are often not tolerable solutions. Fortunately, a host of recent technological developments and techniques have aided in reducing the errors at each stage of treatment from simulation, planning, quality assurance, and delivery [1]. Modern delivery techniques can conform dose fields geometrically to within 2 mm and dosimetrically to within 2-3% [2]. However, this is largely undermined by uncertainty in target definition. Interobserver variability in target segmentation is one of the largest sources of error in radiotherapy [3], with ratios of the largest to smallest gross tumor volume (GTV) definition being as high as 7.66 in lung when using CT alone [4] and 2.63 when using fused PET/CT images [5].

The addition of 2-[18F]-fluoro-2-deoxy-D-glucose(FDG) PET has been shown to provide valuable information to aid in target definition [7] and reduce interobserver variability but suffers from interpatient and interscanner variability and relatively low spatial resolution. For this reason, computer guided delineations based on objective criteria are needed for defining the tumor edge. Most clinically available techniques rely on SUV thresholding choices, of which many have been proposed [8, 9] including tumor volume and background dependent formulas [10–12]. However, thresholding techniques are unable to address confounding factors such as uptake heterogeneity, partial volume effect, and tissue inflammation which can influence interpretation of PET images. Thresholding techniques are thus unable to consistently delineate the tumor volume accurately [13, 14]. A significant number of alternative segmentation techniques have been proposed to provide a more robust delineation of the gross tumor volume (GTV) and biological target volume (BTV). For example, several unsupervised approaches such as the fuzzy locally adaptive Bayesian segmentation approach (FLAB and 3-FLAB) [15, 16] and fuzzy hidden Markov chains (FHMC) [6] assign a fuzzy classification using the voxel environment and noise by iteratively modelling the probability distributions of different classes found within the current image. Fuzzy clustering methods (FCM and FCM-SW) [17, 18] are similar approaches that find distinct groupings within the intensity distributions. FCM-SW is an application of clustering that relies on a handful of preprocessing filters to eliminate the effects of noise and heterogeneity. However, these algorithms usually require an initial class estimate as well as a bounding box, and their implementations have not yet incorporated information from more than one modality. Ultimately, while previous work in this area has been promising, application has so far been limited to first-order statistical features and a small number of spatial features.

An overwhelming amount of additional information is present within PET/CT images in terms of textural and structural characteristics. The use of texture features as a means of automatic segmentation has shown promising results, providing additional information that can improve the robustness of delineation criteria. This has been shown in multiple modalities including ultrasound [19] and MRI [20]. PET and CT textures in the lung have been used in a large number of applications such as differentiating malignant from benign lymph nodes [21, 22], judging treatment response [23], diagnosing diffuse parenchymal lung disease [24–26], determining tumor staging [27], detection [28], and segmentation [29]. However, these applications have used the textures from each modality independently. A number of groups have suggested that the use of information from both PET and CT should provide improved segmentation over the use of either modality alone. Because of this a handful of algorithms exist that utilize the combination of multimodality segmentation such as the multivalued level set (MVLS) [30], the variational Bayes inference [31], and graph based methods [32]. Our group also previously demonstrated that the use of a combination of PET and CT texture features in images of patients with head and neck cancer improved classification versus using PET and CT separately [33]. Similarly, segmentation within the context of lung could benefit from such a strategy, particularly in cases where the tumor is highly integrated into the mediastinum, shows heterogeneous uptake, or is surrounded by collapsed lung or inflamed tissue. Unfortunately, it cannot be assumed that the texture characteristics of abnormal and normal tissues in the head and neck will be consistent with the thorax, hence this paper aims to evaluate this strategy in the lung by (1) characterizing a larger list of texture features in the lung that can or have been shown to be useful for differentiating tumor from healthy tissue in PET/CT images, (2) determining the optimal combination of such features for segmentation, and (3) validating the results against expert observer contours.

## 2. Materials and Methods

### 2.1. Test, Training, and Validation Data

Coregistered FDG-PET/CT scans of 34 cases presenting both small cell and nonsmall cell lung carcinoma (stages T1–T4) and undergoing treatment at the Princess Margaret Hospital in Toronto, ON, were selected for training data. Research ethics board approval and informed consent were acquired for all patients. Scans were acquired prior to treatment. Regions of interest (ROIs) were drawn manually around the gross tumor volume by a radiation oncologist experienced in treatment of lung carcinoma, and healthy structures such as the heart, liver, spleen, trapezius muscle, descending aorta, fat, sternum, spinal cord, and vertebral bodies were contoured afterwards by a graduate student. Tissues were chosen based on two criteria, whether they contained unique attenuation values, or if they typically have high FDG uptake values (i.e., liver and heart), and thus may possibly overlap with tumor FDG uptake distributions. A 7 mm margin was subtracted from the edges of all ROIs to avoid filter artifacts caused by the sudden changes in intensity at the borders of objects from contaminating the feature measurements. Of the 34 cases, noise was estimated using the definition of signal to noise ratio (SNR) as follows:

(1) $$\begin{array}{c} \text{SNR}=\frac{\mu_{\text{liver}}}{\sigma_{\text{liver}}}, \end{array}$$

where *μ* and *σ* are the mean and standard deviation of a ROI drawn around the liver. The liver was chosen because it provided a large, homogeneous region with a significant signal in both the CT and PET images. The ROIs were drawn as to carefully avoid any inhomogeneities due to vasculature or nonfunctioning portions of tissue. In order to prevent noise from contaminating the texture measurements a SNR of 3.7 taken from the CT values was chosen as a cutoff leaving 21 patients remaining for use in training. This was decided visually from the texture maps, where a decrease in the texture map quality was seen past this point. The CT SNR was used since the PET SNR remained fairly consistent with a range of 6.12–10.09, mean of 7.56, and standard deviation of 1.12. 43; feature images in total were calculated for the PET and CT volumes. The features chosen for characterization fall into 5 categories, first order, second order, higher order, structural features, and Tamura features. For each ROI, the feature values were averaged at every slice which constituted the samples. This was done in order to partially account for variability within the patient. In total 2385 samples were calculated of which 337 were abnormal, and 2048 were normal.

Additionally 10 patients presenting with nonsmall cell lung carcinoma and undergoing treatment at the Odette Cancer Centre in Toronto, ON, were chosen to validate the performance of the texture training data for classification of abnormal from normal tissue. The patients selected were deemed difficult to contour manually and hence would possibly showcase the advantages of using texture above alternative measures. In addition it was desired to determine whether the ROI-based training data could be reliably used for voxel-by-voxel segmentation of images taken from a different scanner and institution.

Three radiation oncologists manually contoured GTVs for the 10 test patients to provide a ground truth. The simultaneous truth and performance level estimation (STAPLE) algorithm was used to combine the GTVs into probabilistic maps for each patient. Since this probabilistic map was used as a ground truth for these cases, slightly modified definitions of sensitivity, specificity, and the Dice coefficient, (2), were used. Here *P*(*x*_*i*_ = *T*) and *P*(*x*_*i*_ = *F*) are the probability values given by STAPLE showing that the voxel *x*_*i*_ is belongs to the abnormal or normal class, respectively, *Ω*_−_ and *Ω*_+_ are the subsets of voxels found outside and inside the segment, *Ω* is the full image domain, and |*Ω*| is the volume of the segment. All three oncologists had experience in contouring thoracic tumors. Common guidelines for contouring were agreed upon by all three oncologists prior to contouring. The PET and CT volumes were presented to the oncologists using two sets of three windows, each displaying the PET, CT, and CT with the PET overlaid in a color scale. Two CT window and level settings were used for the two sets in order to simultaneously enhance the tissue to air boundary and subtle soft-tissue contrast. These were window/level settings of 1600/−400 HU and 400/+40 HU, respectively. Because of interpatient SUV variability among both benign and malignant tissue, the SUV window and level settings were left to the discretion of the oncologist, similar to the clinical setting. No additional patient information was provided, as this would be information not used by the automated program and hence would bias results. Any visible positive nodes easily discernible from any adjoining tumor mass were separately contoured. In total 10 primary tumors and 19 positive nodes were contoured as follows:

(2) $$\begin{array}{c} \text{Sensitivity}=\frac{\sum_{i\in \Omega_{+}}P\left(x_{i}=T\right)}{\sum_{i\in \Omega_{+}}P\left(x_{i}=T\right)+\sum_{i\in \Omega_{-}}P\left(x_{i}=T\right)},\\ \text{Specificity}=\frac{\sum_{i\in \Omega_{-}}P\left(x_{i}=F\right)}{\sum_{i\in \Omega_{-}}P\left(x_{i}=F\right)+\sum_{i\in \Omega_{+}}P\left(x_{i}=F\right)},\\ \text{Dice}=\frac{2\left(\sum_{i\in \Omega_{+}}P\left(x_{i}=T\right)\right)}{\sum_{i\in \Omega }P\left(x_{i}=T\right)+\left|\Omega +\right|}. \end{array}$$

The 21 training cases were also utilized for validation by performing a leave-one out training of 21 different DTKNNs and using them to segment the case that was excluded from their training set. For these cases the GTV included in the treatment plan was used for validating the segmentations.

### 2.2. Scanning Parameters and Preprocessing

Training data scans of the thorax were acquired 1 hour after-injection of 5 MBq/kg of FDG using a Discovery ST PET/CT scanner (GE Medical Systems Waukesha, WI) in CINE mode using a CT voxel size of 0.98 ×  0.98 ×  2.5 mm and a PET voxel size of 3.9 × 3.9 × 3.27 mm. PET images were reconstructed using an Ordered Subset Estimation Maximization (OSEM) algorithm. Scatter correction was performed using convolution subtraction, decay was corrected to the acquisition start time, and attenuation correction was performed using the CT volumes. Transaxial matrix sizes were 512 × 512 and 128 × 128 for CT and PET, respectively. An initial ungated CT scan was acquired at 120 kVp with a current of 105 mAs/slice for 0.956 s per slice in helical mode for attenuation correction of the PET image. Then two subsequent gated CT scans at inhale and exhale were acquired at 120 kVp and 180 mAs/slice for 0.5 s per slice. The reconstructed field of view (FOV) was 50 cm for both PET and CT volumes. All PET images were upsampled to match the resolution and size of the CT volume using linear interpolation. If the resulting size of the PET was larger than the CT volume, the PET image was cropped and manually realigned to the CT. The inhale phase CT volume was chosen for feature analysis over the ungated scan, as it provided better image quality and resolved the issue of gating artifacts. However, since the PET volumes were physically coregistered to the ungated CT scan, they were deformed to the inhale phase using deformation vectors calculated by nonrigidly registering the ungated CT to the inhale CT volume using the symmetric log-domain diffeomorphic demons algorithm (ver. 0.0.4) provided by the Insight Journal. All other correction and reconstruction algorithms were vendor supplied.

The test patients were scanned 1 hour after-injection of 5.5 MBq/kg of FDG to a maximum of 370 MBq using a GEMINI PET/CT scanner (Philips Medical Systems, Cleveland OH) with a CT voxel size of 0.98 × 0.98 × 3.0 mm and a PET voxel size of 4.0 × 4.0 × 4.0 mm. PET images were reconstructed using a 3D RAMLA algorithm with scatter correction using a Monte Carlo single-scatter simulation, decay correction using the acquisition start time and attenuation correction using the CT volumes. Transaxial matrix sizes were 512 × 512 and 144 × 144 with a FOV of 45 and 57.6 cm for CT and PET, respectively (a FOV of 60 cm was originally used for the CT when attenuation correction was performed). CT images were acquired at 120 kVP with a current of 250 mAs/slice for 1 s per slice. CT images were scanned in helical mode.

Prior to filtering, the CT image was scaled into 256 bins, while the PET image was scaled using bins of 0.05 SUV. This was done to avoid excessive computation time when calculating the cooccurence matrices for the Haralick texture features.

### 2.3. Feature Calculations

**Figure 1:**
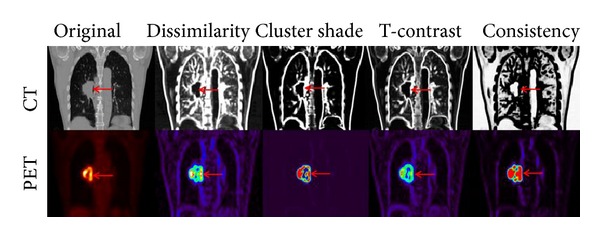
Examples of the different texture maps derived from SGLDM and NGTDM features for coronal CT and PET images of the same patient.

The features chosen for characterization fall into 5 categories. Unless otherwise stated, a 7 × 7 × 3 voxel neighbourhood was used for every feature calculation, as it reflected the anisotropic resolutions of the PET/CT volumes. This was done, so that the physical dimensions of the neighbourhood were as close to being equal as possible. The distance between cooccurring voxels was always chosen to be 1 (any adjoining voxels). Visual maps of the additional features not considered in the work by Yu et al. [33] are shown in Figure 1.

#### 2.3.1. First-Order Histogram Statistics

 These features rely on information derived from single-voxel values and their distribution found within a neighborhood window centered around any given voxel. The measurements are simple statistical characteristics and include mean, median, standard deviation, skewness, and kurtosis. While simple, many algorithms have incorporated the use of these features [6, 18, 34].

#### 2.3.2. Second-Order Features

 Second-order features refer to characteristics that take into account the context by which pairs of voxels are found or their cooccurrence. These features are derived using a spatial grey-level dependence matrix (SGLDM) which is determined by the joint-probability distribution of each combination of grey-level values that occur next to each other, averaged over angles of 0°, 45°, 90°, and 135°. These features were first developed by Haralick et al. [35] in 1973 and have been shown to be useful for classification of aerial, satellite, and photomicrograph images. Features used from this work include energy (i.e., angular second momentum), entropy, sum average, homogeneity, correlation, dissimilarity, and contrast (termed “S-contrast”). An additional feature, cluster shade, formulated by Conners et al. [36] was also included as a measure of the discrepancy in size of homogenous clusters. Additionally a new feature termed “consistency” was created that offers an alternate measure of occurrence homogeneity, as this characteristic has been seen to be characteristic of tumors in PET [33] as follows:

(3) $$\begin{array}{c} \text{Consistency}=\frac{\sum_{i}^{N_{g}}\sum_{j}^{n}P\left(i,j\right)\cos\left[\left(i-j\right)(2\pi /N_{g})\right]}{\sum_{i}^{N_{g}}\sum_{j}^{N_{g}}P\left(i,j\right)}, \end{array}$$

where *P*(*i*, *j*) is the (*i*, *j*)th entry of *N*_*g*_ × *N*_*g*_ cooccurrence matrix, and *N*_*g*_ is the number of discretized grey levels. The basis of using these features is to detect the distinct vascular and structural pattern that is characteristic of cancerous lesions. While there are more second-order features, those included in this category were chosen based on their general response to heterogenous patterns as well as mathematical independence. Second-order features have been well correlated with human perception in textural discrimination studies when no other visual cues were available [37].

#### 2.3.3. Higher-Order Features

 Higher-order texture statistics refer to similar measurements based on the relationship of more than two voxels at a time; here the contrast with the neighborhood grey tone is considered. The features considered were formulated by Amadasum and King in 1989 [38] who proposed the use of Neighborhood Grey-Tone Difference Matrices (NGTDMs) to describe visually intuitive concepts such as coarseness, contrast (referred to here as “N-contrast”), and busyness. While only an approximation of human visual perception of texture, higher-order features can detect subtle statistical differences well beyond that of a human observer. Due to their sensitivity to local variations and independent method of calculation, these texture characteristics were considered to compliment the previously mentioned features. The modified formulations in Yu et al. [33] were used in this case in order to be intensity scale invariant.

#### 2.3.4. Structural Features

 Structural features were included in order to characterize the macrotextures present in the human thoracic anatomy. This is necessary as all the features listed so far are limited to fine scales due to their computational impracticality at larger neighbourhood sizes. Three structural features were chosen in this category, the morphological gradient, the standard deviation of the morphological gradient, and the left-to-right symmetry ratio. The morphological gradient is determined by subtracting the dilation of an image by its erosion and was calculated using the Insight Toolkit (ITK) (Kitware Inc., New York NY), with a ball kernel of radius 2 voxels. The standard deviation of this morphological gradient image was also taken as a feature by scanning a neighborhood window over the image. The left-to-right symmetric ratio was calculated by dividing a given neighborhood of voxels by the same neighborhood mirrored across the left-right axis.

#### 2.3.5. Tamura Features

 Tamura features are an alternative method for computationally approximating perceptual concepts such as coarseness, contrast, and directionality. These features were formulated by Tamura et al. in 1978 [39] and were found to correlate well with visual rankings of texture patterns. Of the 6 features proposed in this work only contrast and directionality were chosen, as they showed the highest correlation with texture rank, were mathematically independent formulations, and lent themselves well to calculation within a limited neighborhood size.

### 2.4. KNN Classifiers and Decision Trees

A K-nearest neighbor or KNN is a simple nonlinear classifier that is able to use prior training data to make predictions regarding new data using the Euclidean distance in feature space. This distance is calculated from (4) for each of the training samples as follows:

(4) $$\begin{array}{c} D_{i}=\sqrt{\sum_{j=1}^{n}{\left(\chi_{j}-x_{ji}\right)}^{2}}, \end{array}$$

(5) $$\begin{array}{c} \text{confidence}=\arg\;\max\left(\frac{n_{a}}{k},\frac{n_{n}}{k}\right). \end{array}$$

Here *χ* is the feature vector of a test point, being classified by its *n*-dimensional distance to the training point *x*_*i*_. To avoid overfitting to the training data and thus ensuring generality, rather than taking the nearest data point, the distances of the *k* closest training examples are used. *n*_*a*_ and *n*_*n*_ are the number of these *k* examples that fall into either class (abnormal or normal). The classification decision is made by choosing the class with the highest number of examples within the *k* examples chosen. In this work a *k* value of 7 was used. A confidence value (5) is determined by the ratio of examples from each class, which was used to calculate the receiver operating characteristic (ROC) curve. KNN classifiers are attractive because of their simplicity and performance which has been found to be competitive with more sophisticated classification algorithms [40, 41]. In order to account for the different scaling of all the features included, each feature value was subtracted from the training mean and divided by the standard deviation. Decision trees are a method used for combining multiple decision making rules in such a way as to partition the training data into multiple sets which can each be classified according to different sets of rules or in this case feature subsets. In previous work, it was shown that a combined decision tree K-nearest neighbors resulted in excellent classification [42]. The training method involves an exhaustive search of at maximum three features for each node that provide the best AUC90 (area under the ROC greater than 90% specificity). The training data is then split using this classifier, and falsely classified examples in both branches are further teased out by training additional KNNs and repeating the process. This tree growing is stopped once either a further improvement in overall classification accuracy cannot be achieved, or the number of training samples in either class fall below a certain number, in this case 4 to be able to achieve a majority out of 7. AUC90 was chosen as it was more sensitive to the shape of the ROC curve near the plateau edge. The ROC was determined by performing a leave one out cross-validation of the ROI-based training data and using the confidence value given in (5) to provide multiple points.

**Figure 2:**
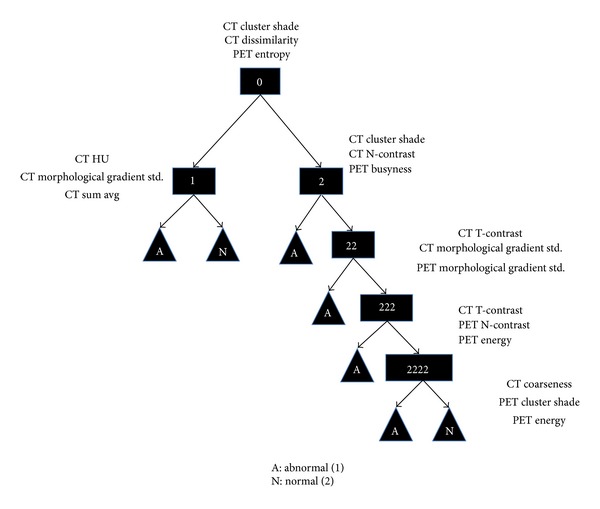
The trained DTKNN tree along with the three best features chosen for each node. Note that this DTKNN was trained to discriminate between the tumor ROIs defined by an oncologist and the normal tissue ROIs defined by a medical physicist for a set of 21 patient PET/CT images.

**Figure 3:**
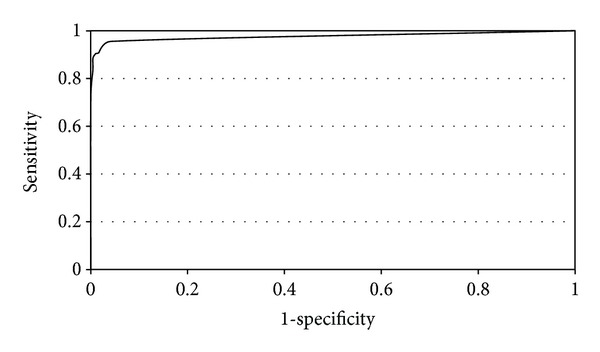
The receiver operating curve for the DTKNN tree using the *k* value determined at the leaves of the tree as the threshold. Note that this ROC shows the ability of the DTKNN to distinguish tumor ROIs from normal tissue ROIs in the training set.

The resulting tree is shown in Figure 2. It consists of 5 levels, with all but one containing a combination of PET and CT features, suggesting that the combination of the two modalities generally provides a better classification. The resulting tree was able to classify the training data ROIs with an AUC of 0.996, an AUC90 of 0.097, and a sensitivity and specificity of 0.973 and 0.991, respectively. The ROC was calculated using a leave-one-out method where each data point is left out of the training data, while it is being classified. The sensitivity and specificity are listed where a *k* value of 4 or greater is required to select either class in the final leaf of the tree. The ROC is shown in Figure 3.

### 2.5. Segmentation Pre- and Postprocessing

Extending ROI-based training results to voxel-by-voxel segmentation in an independent set of images required additional processing steps. In order to reduce computation time and improve accuracy of the segmentation, several steps were taken prior and after classification. Only voxels with an SUV greater than 1, a HU value between −300 and 200, and a PET busyness that did not equal zero and was less than 0.8 were considered. This automatically removed voxels from regions that had little FDG uptake as well as nonsoft tissue from consideration. Thresholding PET busyness was done to remove many heart and liver voxels from consideration. Connected component analysis was performed after segmentation to remove classification errors which were characterized as sparse voxels distributed throughout the volume. Components with a volumes less than 0.5 cm^3^ were reclassified as normal as these were thought unlikely to represent actual tumor. Contours were then dilated by a disc of radius 2 voxels, sent through a morphological closing, then a fill procedure, then eroded back to the correct size by the same disc. The CT window chosen in preprocessing was then reapplied to eliminate any voxels that may have accidentally leaked into bone or air. This last series of steps was performed because the algorithm would occasionally miss sections at the center of the tumor where FDG uptake was fairly homogenous. This occurred due to the fact that many of the features look for sharp gradients in FDG uptake.

### 2.6. Thresholding Methods

A SUV threshold of 2.5 proposed by Paulino et al., [9] and thresholds of 20%, 25%, 30%, 35%, 40%, and 50% of the maximum uptake value were included in a comparison. It was found that among the various percentages, the 30% max SUV threshold performed the best according to Dice coefficient, and hence for brevity only this threshold is included in the results section.

### 2.7. First-Order KNN

In order to see whether the addition of feature information played a significant role in segmentation, a single KNN classifier using only SUV and HU was included for comparison. This is referred to as “HU-SUV-KNN” in Figures 6, 7, and 8.

### 2.8. 3 Class Fuzzy Locally Adaptive Baysian (3-FLAB)

The 3-FLAB algorithm developed by Hatt et al. [16] was implemented to enable comparison against a current state-of-the-art, PET-only algorithm. In this algorithm a fuzzy classification is performed between three hard classes. A fuzzy K-means algorithm is used to initialize stochastic estimation maximization. An iterative estimation of the probability of each voxel's class is based on its 3 × 3 × 3 neighbourhood in order to improve robustness to noise.

### 2.9. Fuzzy Clustering Method

The FCM algorithm included in the fuzzy logic toolbox of MATLAB was used to cluster the PET data into three classes as a means of segmentation. The class with the largest resemblance to the tumor boundary as defined by the oncologist was chosen and thresholded at a class membership probability of 50%.

### 2.10. Multivalued Level Set Method

The multivalued level set algorithm as published by El Naqa et al. in 2007 [30] is a geometric variational method that incorporates multimodal information using a set of weights to emphasize the importance of each force evolving the contour and each modality. The level set method begins with an initialization and represents the contour implicitly using a level set function where the contour is defined by the zero crossing of this function. Here the metric is defined by (6) which relies on the differences in mean intensity inside and outside the segment and is maximized through iterative evaluation of the function gradient as follows:

(6) infCJ(C,c+,c−)∝μ,length(C)+1N∑iλi+∫Ω|Ii−ci+|2H(ϕ)dx+λi−  ×∫Ω|Ii−ci−|2(1−H(ϕ))dx+1N∑iγi(ci+−ci−)2,

where *C* is the contour, *c* ^+^ and *c* ^−^ are the regions outside and inside the contour, *N* is the number of samples taken from the images indexed by *i*, *ϕ* is the scalar level set function, and *H*(*ϕ*) is the Heaviside function. *Ω* is the image space, and *λ*_*i*_^+^, *λ*_*i*_^−^, and *γ*_*i*_ are the weights attributed to the outside, inside and spring force of the equation for input *i*. *μ* is another weighting factor associated with the length of the contour, controlling the smoothness of the segment. Due to the fact that active contour methods are generally sensitive to the initial segmentation, two methods of initialization were used. The first method used the physician contours, following erosion using a square structuring element with a width and height of 4 voxels applied two-dimensionally to every slice. In the case of the testing data when using the STAPLE derived probabilities, this erosion was applied to the binary mask of the 90% threshold. The second method calculated the center of mass for each connected component of the binary mask defining the manual delineations. The binary mask taken by thresholding the 90% probability level in the STAPLE maps was similarly used for the 10 test patients. Spheres with a radius of 8.16 mm or 8 voxels in the transverse plane, centered on the centers of mass of the connected components, were then used as initialization. The first method is referred to as the manual method in the results and was included to demonstrate the optimal results that the MVLS could achieve. The second is referred to as the spheres method and was included to not only show how initialization would effect the results but also to replicate the method by which the algorithm was initialized in the paper by El Naqa et al. [30]. The parameters chosen were *λ*_*i*_^+^, *λ*_*i*_^−^, and *γ*_*i*_ equal to [1, 1.5], [1, 1.5], and [0.01, 0.015] where the first element refers to the PET weight and the second to the CT. For three cases in the training data where instability was observed, the weights were changed to [1.5, 1], [1.5, 1], and [0.2, 0.3] in order to rely more heavily on the PET image and spring term, *γ*. A step size of 0.05 and a maximum iteration limit of 250 steps were used in order to provide a smooth evolution of the level set function and ensure that convergence was reached. A *μ* value of 0.5 was used.

## 3. Results

**Figure 4:**
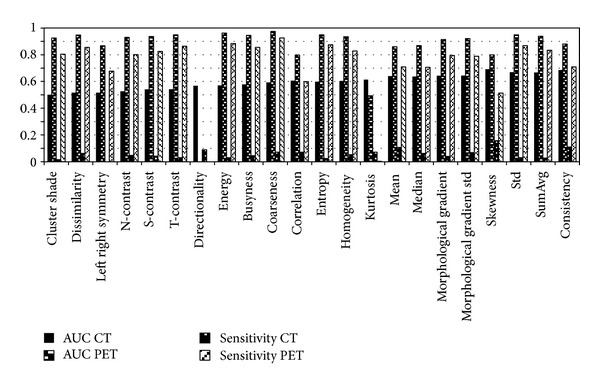
Performance characteristics of the chosen features in terms of area under the curve (AUC) and sensitivity. Specificity was excluded as all values were above 0.95 and showed little difference between features. Directionality was also excluded for application to the PET images and is thus not shown.

**Figure 5:**
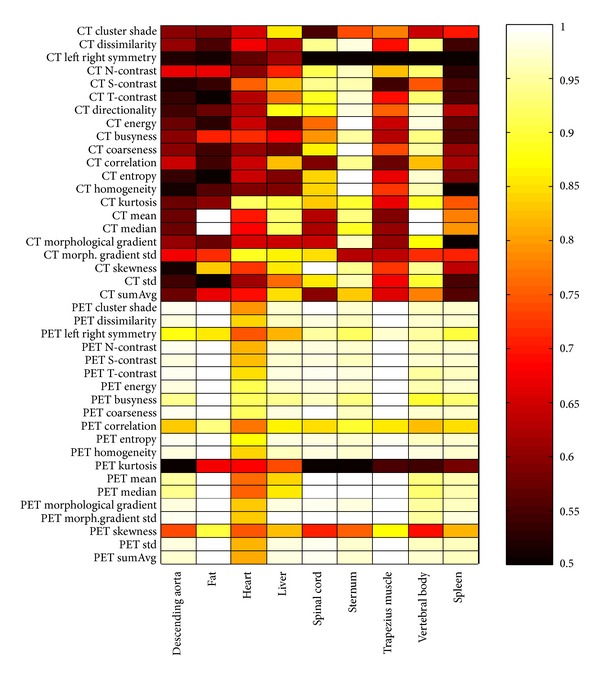
Summary of performance by area under the receiver operation characteristic (ROC) curve (AUC) when using individual features to discern a given tissue from the tumor volume. The *k* value of the KNN classifier was used to vary sensitivity and specificity and plot the curve. Lighter areas indicate better discrimination. Note that these data refer to discrimination of tumor ROIs from normal tissue ROIs, not individual voxel discrimination.

The individual classifier results in Figures 4 and 5 show a great disparity between the CT and PET features, which is to be expected considering the sensitivity of FDG. The most discriminatory PET and CT features were CT skewness and PET coarseness with AUCs of 0.692 and 0.975, respectively, for discriminating between tumor and normal tissue ROIs.

In order to judge their potential for combinatorial strategies, their performance with regards to discrimination of individual tissue types was also evaluated and summarized in Figure 5. Here the AUC is shown on a warm color scale ranging from 0.5 to 1. The tiles show the performance of the feature for discriminating individual organ ROIs from tumor ROIs.

Each feature was also evaluated for its robustness to noise. To do this, the Pearson's correlation coefficient was calculated between the mean liver ROI feature value, and the SNR was calculated from the same ROI. The majority of PET features showed little correlation to SNR, while some of the CT features showed high correlation, suggesting susceptibility to noise. CT skewness showed the least correlation with an *r* value of 0.06, while most of the SGLDM and NGTDM CT features showed a much higher correlation with SNR.

**Figure 6:**
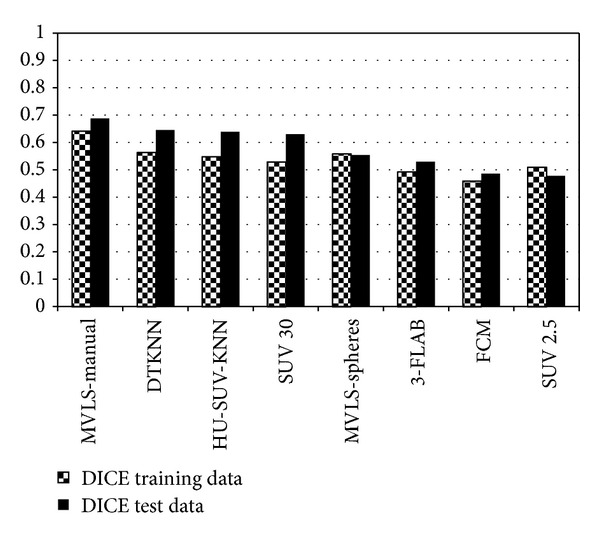
Summary of the average Dice coefficient for both datasets. The ground truth was evaluated using a single physician contour for the training data and an estimated consensus using STAPLE for the test data which explains the generally higher scores for each algorithm.

**Figure 7:**
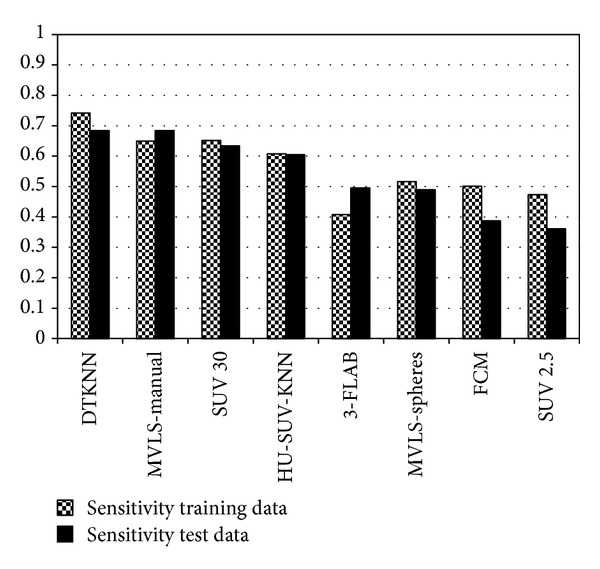
Summary of the average sensitivity for each algorithm. The DTKNN algorithm showed the highest sensitivity to abnormal voxels with an average value of 73.9%.

**Figure 8:**
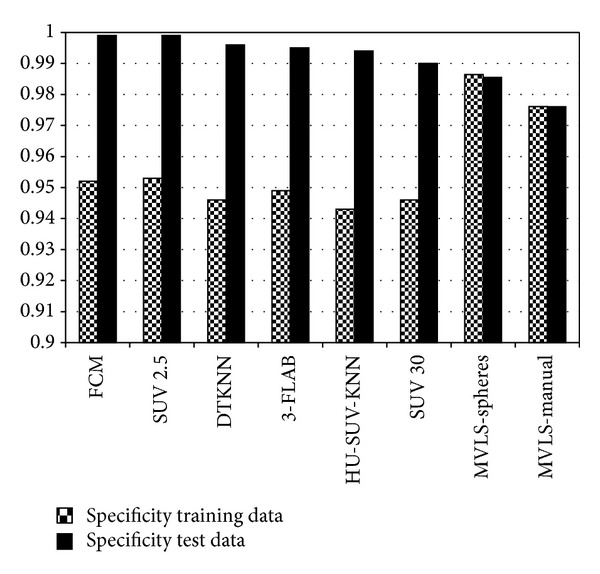
The average specificity for each algorithm. The majority of the tested algorithms had a higher specificity for the test data following the trend of the Dice coefficients with the exception of the MVLS method.

**Figure 9:**
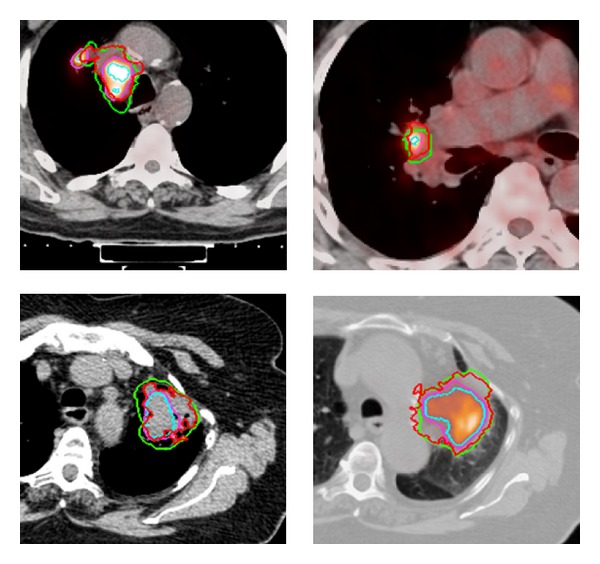
Results from the voxel-by-voxel DTKNN classification (red) shown with the oncologist's GTV contour (green) overlaid on transverse slices of a fused PET/CT volumes (top left, top right, and bottom right). The highest performing PET threshold of 30% max SUV (purple) is also shown overtop of the best performing hard class defined by the 3-FLAB algorithm (teal).

Using the probability maps calculated by STAPLE and the GTVs of the 21 training patients, the voxel-by-voxel performance of the various segmentation methods, including the DTKNN, two threshold techniques, a single KNN based on Hounsfield units (HU) and SUV (referred to as HU-SUV-KNN), MVLS, 3-FLAB, and the fuzzy clustering method was evaluated. This is demonstrated for 4 cases in Figure 9 using the DTKNN, a manual contour, a threshold of 30% SUV and 3-FLAB. Three metrics were used, Dice coefficient, sensitivity, and specificity. The DTKNN was shown to have the second highest average Dice coefficient across both data sets of 0.607. When validated among the training data alone, the classifier performed with an average Dice coefficient, sensitivity, and specificity of 0.571, 0.742, and 0.946, respectively. Similarly when validated using solely the 10 independent patient images the classifier was found to perform with the same respective metrics at 0.654, 0.684, and 0.996 as shown in Figures 6, 7, and 8. The DTKNN algorithm performed with the highest sensitivity compared to other segmentation methods but had one of the lowest specificities due to false positive regions such as the left ventricle of the heart, superior edge of the liver, and a slight overestimation of the tumor boundary. The concordance index (CI) of the three observers was calculated according to the definition by Struikmans et al. [43]. The average CI of the 10 test patients from Sunnybrook was found to be 0.588. Since variability based on training data is a concern for decision trees [44], a cross-validation leave one out of each training patient was performed. For each leave one out case, the DTKNN was retrained on the remaining data and the resulting tree, and data set was used for segmentation of the 10 test cases. The standard deviation among the Dice coefficients for each leave one out case was calculated for each test patient. The average value of this as a percentage of the average Dice coefficient was found to be 14%.

The MVLS-manual method was shown to perform with the highest average Dice coefficient for both datasets and the second highest sensitivity when using the manual method. However, these optimal segmentations would likely not occur consistently in a real world situation, since it relied on data the user might not agree with. When using a consistent initialization using spheres, similar to that used in the paper by El Naqa et al. [30] it was found that the algorithm converged on solutions that did not agree nearly as well with the expert observers' segmentations in terms of Dice coefficient. The MVLS, initialized using spheres, had an average Dice coefficient of 0.590, which was very similar to the average coefficient of the DTKNN (0.607).

## 4. Discussion

Presented in this work is the training and validation of a DTKNN classifier algorithm based on the use of 3D texture features derived from PET/CT scans of patients with lung carcinoma, in order to estimate the contours of the gross tumor volume. The DTKNN differentiates itself from other methods in that it utilizes machine learning to classify voxels as being either normal or abnormal by relying on the textural patterns from both PET and CT. The other algorithms presented in this paper do not rely on previously observed data and only work with knowledge of the case they are applied to. This work develops the DTKNN method further by investigating the use of additional texture features to improve the performance of the algorithm and characterizes these features in the context of the lung.

One of the challenges of validating image segmentation is determining a ground truth. While the use of contours derived from histological examination of surgically resected tumors would seem an ideal gold standard, errors are often introduced due to changes in tissue morphology during resection, histoprocessing [45], and during registration [46]. While a great deal of interobserver variability exists, the use of STAPLE to draw a probabilistic map has shown validity for predicting expert consensus [47] and providing ground truth in the case of the Cardiac Atlas Project [48]. This is combined with the fact that our observers agreed with a concordance index similar to that observed in the study by Struikmans et al., [43] that leads us to believe the STAPLE method was able to provide a reliable ground truth for our test data. It can be seen in Figure 6 that all algorithms performed better with regards to the test data as opposed to the training data, which is indicative of a more reliable ground truth.

To study tree stability, 21 different decision trees were trained via a leave one out approach for each of the patient image sets. The structures of the trees were found to vary widely near the terminal ends. In terms of the classification results, standard deviation as a percentage of the average Dice coefficient was 14%, which is not unreasonable; however, this suggests that the method may benefit from a larger training set.

One of the drawbacks encountered in the preprocessing of our method were in conditions where a tumor may present a necrotic core with a low uptake. In a few instances, this produced a small cavity in the segmentation which is another reason why the fill procedure was included in the postprocessing step. However, if the segmentation is not a closed shape, this would of course not work.

The performance results showed that the DTKNN segmentations produced a statistically significant (*P* < 0.05) improvement in sensitivity compared to the tested algorithms when using a Student *t*-test. Improvement in Dice coefficient was statistically significant when compared to all algorithms with the exception of the 30% max SUV threshold and the MVLS algorithm. However, the literature and the results of this work have shown the 30% SUV threshold and thresholding in general to be an inconsistent segmentation technique [13, 14]. The DTKNN did not show the highest specificity which suggests overestimation of the boundaries. However, this may be preferable to an underestimation due to the dangers of recurrence from undertreating the target. While the DTKNN algorithm did show a slightly higher average Dice coefficient than the MVLS when initialized using spheres, it cannot be concluded that there was a significant difference in performance between the DTKNN and the MVLS using this dataset.

It was found that all three performance metrics were drastically changed by the choice of initialization for the MVLS. The DTKNN has an advantage in this regard in that it does not require user input.

From Figure 5 we can see that while the general performance of some individual features may be poor, there is a considerable amount of interplay that may be exploited between them. A large number of CT features can be seen to have difficulty in distinguishing abnormal tissue from the descending aorta and spleen. Unsurprisingly the organ in which the PET features had the lowest performance was with the heart due to frequent cases of high myocardial glucose utilization in the training data and hence high uptake values.

When combining these features using a DTKNN for classification it was found that the resulting classifier could outperform a number of alternative segmentation methods in terms of Dice coefficient and sensitivity. The HU-SUV-KNN classifier was included to determine whether the inclusion of statistical, structural, and textural features offered an improvement to using the unprocessed intensity values alone. The improvement in Dice coefficient showed that the addition of texture improved the overlap of the segmentation with the ground truth. The results also suggest that the performance remains relatively consistent across images taken from a different scanner and institution when compared to alternative segmentation methods. It must be noted that the DTKNN, HU-SUV-KNN, and MVLS methods were the only ones tested to incorporate information from both PET and CT in their segmentation. It would also appear that for the most part, these methods performed significantly better than the PET—only algorithms included in this study. If CT features were added to some of the other methods, it seems likely that their segmentation performance might also be improved. It should be noted, however, that with the inclusion of a second modality into the segmentation process, registration errors will inevitably also be introduced. From the results shown here, it would seem that the benefit to accuracy outweighs the additional error. The inclusion of additional modalities such as magnetic resonance images may be able to further improve results; however, the intuition and anatomical knowledge that an expert observer can possess will always remain a challenge to replicate using automated and semiautomated software.

Future work could involve the incorporation of 4D-PET images in order to better coincide with the gated CT images and reduce motion blurring. Whether 4D-PET holds useful texture information and whether the texture itself demonstrates dynamic properties is still unclear.

## 5. Conclusion

 Presented is a characterization and usage of 3-dimensional texture features found within the thorax for segmentation of carcinoma in coregistered PET/CT scans. The approach relies on a DTKNN to classify voxels based on their first-, second-, and higher-order statistics as well as structural and Tamura features. Of these it was found that, independently, CT skewness and PET coarseness were the strongest discriminators of carcinoma from healthy tissue for each modality with AUCs of 0.692 and 0.975, respectively. For validation, contours of 10 test patients deemed difficult to contour from 3 oncologists were combined into a probabilistic map using STAPLE along with the 21 training images which were evaluated using a leave-one-out method. Utilizing the features in conjunction within the DTKNN it was found that the tree could outperform a variety of threshold methods, an implementation of the 3-FLAB algorithm, and a single KNN based on Hounsfield units and standard uptake value alone for defining the tumor volume. The approach was able to segment tumor with an average Dice coefficient of 0.607 and an average sensitivity and specificity of 73.9% and 99.2% across both data sets, respectively. The results show that the usage of texture features within PET/CT images of the thorax is a promising approach for target delineation in radiotherapy of the lung, automatically producing GTVs both qualitatively and quantitatively similar to a consensus of expert observers.

## References

[1] Haasbeek CJA, Slotman BJ, Senan S (2009). Radiotherapy for lung cancer: clinical impact of recent technical advances. *Lung Cancer*.

[2] Li XA (2011). *Adaptive Radiation Therapy*.

[3] Weiss E, Hess CF (2003). The impact of gross tumor volume (GTV) and clinical target volume (CTV) definition on the total accuracy in radiotherapy: theoretical aspects and practical experiences. *Strahlentherapie und Onkologie*.

[4] van de Steene J, Linthout N, de Mey J (2002). Definition of gross tumor volume in lung cancer: inter-observer variability. *Radiotherapy and Oncology*.

[5] Caldwell CB, Mah K, Ung YC (2001). Observer variation in contouring gross tumor volume in patients with poorly defined non-small-cell lung tumors on CT: the impact of ^18^FDG-hybrid PET fusion. *International Journal of Radiation Oncology Biology Physics*.

[6] Hatt M, Lamare F, Boussion N (2007). Fuzzy hidden Markov chains segmentation for volume determination and quantitation in PET. *Physics in Medicine and Biology*.

[7] Mah K, Caldwell CB, Ung YC (2002). The impact of 18 FDG-PET on target and critical organs in CT-based treatment planning of patients with poorly defined non-small-cell lung carcinoma: a prospective study. *International Journal of Radiation Oncology Biology Physics*.

[8] Erdi YE, Mawlawi O, Larson SM (1997). Segmentation of lung lesion volume by adaptive positron emission tomography image thresholding. *Cancer*.

[9] Paulino AC, Koshy M, Howell R, Schuster D, Davis LW (2005). Comparison of CT- and FDG-PET-defined gross tumor volume in intensity-modulated radiotherapy for head-and-neck cancer. *International Journal of Radiation Oncology Biology Physics*.

[10] Black QC, Grills IS, Kestin LL (2004). Defining a radiotherapy target with positron emission tomography. *International Journal of Radiation Oncology Biology Physics*.

[11] Daisne JF, Sibomana M, Bol A, Doumont T, Lonneux M, Grégoire V (2003). Tri-dimensional automatic segmentation of PET volumes based on measured source-to-background ratios: influence of reconstruction algorithms. *Radiotherapy and Oncology*.

[12] Nehmeh SA, El-Zeftawy H, Greco C (2009). An iterative technique to segment PET lesions using a Monte Carlo based mathematical model. *Medical Physics*.

[13] Biehl KJ, Kong FM, Dehdashti F (2006). 18F-FDG PET definition of gross tumor volume for radiotherapy of non-small cell lung cancer: is a single standardized uptake value threshold approach appropriate?. *Journal of Nuclear Medicine*.

[14] Schinagl DAX, Vogel WV, Hoffmann AL, van Dalen JA, Oyen WJ, Kaanders JHAM (2007). Comparison of five segmentation tools for 18F-fluoro-deoxy-glucose-positron emission tomography-based target volumedefinition in head and neck cancer. *International Journal of Radiation Oncology Biology Physics*.

[15] Halt M, Rest CCL, Turzo A, Roux C, Visvikis D (2009). A fuzzy locally adaptive Bayesian segmentation approach for volume determination in PET. *IEEE Transactions on Medical Imaging*.

[16] Hatt M, Rest CCL, Descourt P (2010). Accurate automatic delineation of heterogeneous functional volumes in positron emission tomography for oncology applications. *International Journal of Radiation Oncology Biology Physics*.

[17] Bezdek JC, Hall LO, Clark MC, Goldgof DB, Clarke LP (1997). Medical image analysis with fuzzy models. *Statistical Methods in Medical Research*.

[18] Belhassen S, Zaidi H (2010). A novel fuzzy C-means algorithm for unsupervised heterogeneous tumor quantification in PET. *Medical Physics*.

[19] Mohamed SS, Youssef AM, Saadany EFE, Salama MMA (2005). Artificial life feature selection techniques for prostrate cancer diagnosis using TRUS images. *Lecture Notes in Computer Science*.

[20] Woods BJ, Clymer BD, Kurc T (2007). Malignant-lesion segmentation using 4D co-occurrence texture analysis applied to dynamic contrast-enhanced magnetic resonance breast image data. *Journal of Magnetic Resonance Imaging*.

[21] McNitt-Gray MF, Hart EM, Wyckoff N, Sayre JW, Goldin JG, Aberle DR (1999). A pattern classification approach to characterizing solitary pulmonary nodules imaged on high resolution CT: preliminary results. *Medical Physics*.

[22] Silva AC, Paiva AC, Carvalho PCP, Gattass M Semivariogram and SGLDM methods comparison for the diagnosis of Solitary Lung Nodule.

[23] El Naqa I, Grigsby PW, Apte A (2009). Exploring feature-based approaches in PET images for predicting cancer treatment outcomes. *Pattern Recognition*.

[24] Uppaluri R, Hoffman EA, Sonka M, Hartley PG, Hunninghake GW, McLennan G (1999). Computer recognition of regional lung disease patterns. *American Journal of Respiratory and Critical Care Medicine*.

[25] Kauczor HU, Heitmann K, Heussel CP, Marwede D, Uthmann T, Thelen M (2000). Automatic detection and quantification of ground-glass opacities on high-resolution CT using multiple neural networks: comparison with a density mask. *American Journal of Roentgenology*.

[26] Chabat F, Yang GZ, Hansell DM (2003). Obstructive lung diseases: texture classification for differentiation at CT. *Radiology*.

[27] Ganeshan B, Abaleke S, Young RCD, Chatwin CR, Miles KA (2010). Texture analysis of non-small cell lung cancer on unenhanced computed tomography: initial evidence for a relationship with tumour glucose metabolism and stage. *Cancer Imaging*.

[28] Saradhi GV, Gopalakrishnan G, Roy AS A framework for automated tumor detection in thoracic FDG PET images using texture-based features.

[29] Zhou J, Chang S, Metaxas DN, Zhao B, Ginsberg MS, Schwartz LH An automatic method for ground glass opacity nodule detection and segmentation from CT studies.

[30] El Naqa I, Yang D, Apte A (2007). Concurrent multimodality image segmentation by active contours for radiotherapy treatment planning. *Medical Physics*.

[31] Wang J, Xia Y, Wang J, Feng DD Variational Bayes inference based segmentation of heterogeneous lymphoma volumes in dual-modality PET-CT images.

[32] Han D, Bayouth J, Song Q (2011). Globally optimal tumor segmentation in PET-CT images: a graph-based co-segmentation method. *Information Processing in Medical Imaging*.

[33] Yu H, Caldwell CB, Mah K, Mozeg D (2009). Coregistered FDG PET/CT-based textural characterization of head and neck cancer for radiation treatment planning. *IEEE Transactions on Medical Imaging*.

[34] Zhang Y, Brady M, Smith S (2001). Segmentation of brain MR images through a hidden Markov random field model and the expectation-maximization algorithm. *IEEE Transactions on Medical Imaging*.

[35] Haralick RM, Shanmugam K, Dinstein I (1973). Textural features for image classification. *IEEE Transactions on Systems, Man and Cybernetics*.

[36] Conners RW, Trivedi MM, Harlow CA (1984). Segmentation of a high-resolution urban scene using texture operators. *Computer Vision, Graphics, & Image Processing*.

[37] Julesz B, Gilbert EN, Shepp LA, Frisch HL (1973). Inability of humans to discriminate between visual textures that agree in second order statistics: revisited. *Perception*.

[38] Amadasun M, King R (1989). Textural features corresponding to textural properties. *IEEE Transactions on Systems, Man and Cybernetics*.

[39] Tamura H, Mori S, Yamawaki T (1978). Textural features corresponding to visual perception. *IEEE Transactions on Systems, Man and Cybernetics*.

[40] Zheng B, Lu A, Hardesty LA (2006). A method to improve visual similarity of breast masses for an interactive computer-aided diagnosis environment. *Medical Physics*.

[41] Christodoulou CI, Pattichis CS, Pantziaris M, Nicolaides A (2003). Texture-based classification of atherosclerotic carotid plaques. *IEEE Transactions on Medical Imaging*.

[42] Yu H, Caldwell C, Mah K (2009). Automated radiation targeting in head-and-neck cancer using region-based texture analysis of PET and CT images. *International Journal of Radiation Oncology Biology Physics*.

[43] Struikmans H, Wárlám-Rodenhuis C, Stam T (2005). Interobserver variability of clinical target volume delineation of glandular breast tissue and of boost volume in tangential breast irradiation. *Radiotherapy and Oncology*.

[44] Litman DJ, Passonneau RJ Combining multiple knowledge sources for discourse segmentation.

[45] Dahele M, Hwang D, Peressotti C (2008). Developing a methodology for three-dimensional correlation of PETCT images and whole-mount histopathology in non-small-cell lung cancer. *Current Oncology*.

[46] Orczyk C, Mikheev A, Rosenkrantz A, Melamed J, Taneja SS, Rusinek H Imaging of prostate cancer: a platform for 3D co-registration of in-vivo MRI ex-vivo MRI and pathology. http://spiedigitallibrary.org/.

[47] Allozi R, Li XA, White J (2010). Tools for consensus analysis of experts’ contours for radiotherapy structure definitions. *Radiotherapy and Oncology*.

[48] Suinesiaputra A, Cowan BR, Finn JP (2012). Left ventricular segmentation challenge from cardiac MRI: a collation study. *Lecture Notes in Computer Science*.

